# The relevance of moral norms in distinct relational contexts: Purity versus harm norms regulate self-directed actions

**DOI:** 10.1371/journal.pone.0173405

**Published:** 2017-03-09

**Authors:** James A. Dungan, Alek Chakroff, Liane Young

**Affiliations:** Department of Psychology, Boston College, MA, United States of America; Mälardalen University, SWEDEN

## Abstract

Recent efforts to partition the space of morality have focused on the descriptive content of distinct moral domains (e.g., harm versus purity), or alternatively, the relationship between the perpetrator and victim of moral violations. Across three studies, we demonstrate that harm and purity norms are relevant in distinct relational contexts. Moral judgments of purity violations, compared to harm violations, are relatively more sensitive to the negative impact perpetrators have on themselves versus other victims (Study 1). This pattern replicates across a wide array of harm and purity violations varying in severity (Studies 2 and 3). Moreover, while perceptions of harm predict moral judgment consistently across relational contexts, perceptions of purity predict moral judgment more for self-directed actions, where perpetrators violate themselves, compared to dyadic actions, where perpetrators violate other victims (Study 3). Together, these studies reveal how an action’s content and its relational context interact to influence moral judgment, providing novel insights into the adaptive functions of harm and purity norms.

## Introduction

Moral judgment applies to a wide array of actions–harmful actions that cause victims physical pain [1–3], as well as impure actions that appear unnatural or elicit reactions of disgust [4,5]. Recent efforts to carve human morality at its cognitive joints have focused on the descriptive content of distinct moral domains (e.g., harm versus purity) [6,7], the relationship between perpetrator and victim (e.g., transgressing against one’s father versus one’s friend) [8–10], and the computational rules that govern judgments across moral domains (e.g., the role of intentions for harm versus purity judgments) [11,12]. Here, we build on this prior work by establishing novel contextual influences on the relevance of moral norms, focusing on the comparison between harm and purity norms as a case study. We demonstrate that moral judgments reflect an interaction between an action’s content and its relational context: moral judgments of purity violations, compared to harm violations, are relatively more sensitive to the negative impact agents have on themselves versus other victims.

Recent work reveals important differences in the cognitive processes underlying moral judgments of harm versus purity violations (for reviews, see [13,14]). For example, people have trouble envisioning circumstances that potentially mitigate the moral wrongness of purity violations compared to harm violations [15]. Other work has shown that contextual features such as whether a violation was committed intentionally or accidentally modulate emotional reactions of disgust in response to purity violations less than anger in response to harm violations [15–17]. Similar patterns have been found for explicit moral judgments: participants perceive a smaller difference between intentional and accidental purity violations (delivering particularly harsh judgments of accidental purity violations) than between intentional and accidental harm violations [11,12]. Recent work has revealed this same basic pattern across small-scale societies [18]. Moreover, brain regions that represent information about intentions (e.g., the right temporoparietal junction) are recruited more for moral judgments of harm versus purity violations and also code (in their spatial pattern of activity) whether harms are intentional or accidental, but not whether purity violations are intentional or accidental [19]. Finally, people make strong person-based, dispositional attributions about purity violators, even when situational factors causing the violation are stipulated (e.g., someone is forced at gunpoint to eat human flesh), whereas people judge situational factors to be the primary cause of harm violations [20]. Together, these findings demonstrate that, while moral judgments of harm violations are sensitive to many contextual features, including the intent of the violator, moral judgments of purity violations are relatively inflexible, tied primarily to the presence of an impure action or outcome.

Given this evidence, an open question is *why* judgments of purity violations are so inflexible relative to judgments of harm violations. On one account, this difference stems from the different relational contexts to which harm and purity norms apply. Harm norms apply primarily to interpersonal or dyadic contexts, where one person’s actions negatively impact another. Indeed, at least two parties–a violator who acts on a victim–may be necessary to establish an act as harmful in the first place (i.e., “stealing” from oneself is simply not stealing) [8,21]. People readily empathize with the pain and suffering of victims [22,23], and even young children spontaneously help others in need [24]. In some cases, people will even harm themselves if doing so protects others from harm [25]. If harm norms dictate how people ought to treat one another, it makes sense that mitigating circumstances about the situation or the violator’s intent (e.g., Jason punched Bob because Bob slept with Jason’s wife; Susan didn’t know that she sent Liz a computer virus) play a significant role in judgments of harm violations: intent information enables accurate predictions of social partners’ future behavior as well as meaningful evaluations of their past and present behavior. That is, people need to understand others’ mental states and situations to evaluate their actions and, importantly, to identify their intentions toward their peers and themselves.

Conversely, purity norms may apply primarily to self-directed actions, in which people act on themselves. Moral psychologists theorize that purity norms have evolutionary roots in disgust responses [26] as part of a mechanism for ensuring people avoid rotten, contaminated food, or otherwise unsafe substances (e.g., feces) [15,27]. These disgust responses may have been co-opted to signal socially and morally offensive behavior (e.g., drug abuse, sexual deviance) [4,28–31]. In other words, the very mechanisms that help people avoid substances that cause disease became useful in identifying people whose non-normative appearance or behavior signals an increased risk of pathogen transmission [32–34]. Importantly, whether purity norms limit the substances people ingest [27,35], the sexual acts they engage in [31,34], or the people they associate with [32–34,36], purity norms protect people for their own benefit. Indeed, people may be concerned with another person’s impurity only to the extent that they feel their own purity is being threatened by the other’s behavior [36–38]. Moreover, and in stark contrast to harmful actions, impure actions are sometimes prohibited even when no one, expect for possibly one’s own self, is rendered a “victim” [5,39]. This account may explain why people care less about the situation surrounding an impure act. As Appiah [40] states in an account of Akran society in Ghana: “With taboo breaking… it doesn’t matter what you meant to do. You’re polluted. You need to get clean” (p. 51). In other words, since purity violations affect oneself, people care less about context and more about simply avoiding the unsavory outcome.

Our recent work suggests that the relational context of moral violations (dyadic versus self-directed) is indeed a critical determinant of whether actions are perceived as harm or purity violations in the first place: dyadic acts are seen as more harmful than impure, whereas self-directed acts are seen as more impure than harmful [11]. However, the question remains of whether or not an action’s relational context interacts with its content to determine moral judgment. The current research provides a direct investigation of this question, predicting systematic differences in people’s moral judgments of harm and purity violations in self-directed versus dyadic contexts. Specifically, our account predicts that for dyadic actions where one person acts on another, harm violations are seen as morally worse than purity violations; in contrast, for self-directed actions where people act on themselves, purity violations are seen as morally worse than harm violations.

We present three studies testing the relevance of harm and purity norms in different relational contexts. Stimuli and dependent measures from all studies are presented in full in Supporting Information (S1 Appendix). To foreshadow the results, in Study 1, we demonstrate that moral judgments of harm and purity violations depend on both the content and the target of the violation: purity violations are more morally wrong than harm violations when they target oneself, but not when they target other people. Studies 2 and 3 replicate this interaction across a wide array of harm and purity violations varying in severity. Study 3 also manipulates both the perpetrator and the victim of violations to test moral judgments of four different relational contexts, finding that perceptions of impurity predict moral judgment more when an action is self-directed than dyadic, whereas perceptions of harm influence moral judgments consistently across relational contexts. Together, these results characterize how both an action’s content and relational context influence moral judgment, providing novel insight into the adaptive function of harm and purity norms.

## Study 1: Harm and purity to self and other

As a first test of our account, we examined two distinct relational contexts: actions targeting oneself versus actions targeting another person. Participants responded to a scenario describing one of four hypothetical actions where participants: (1) harm themselves, (2) make themselves impure, (3) harm another person, and (4) make another person impure.

### Method

Using Amazon Mechanical Turk (MTurk; http://www.mturk.com/), we collected data from an online sample of 526 American participants (no other demographic data was collected). All participants gave written informed consent and were paid $0.10 for their time. All experimental procedures were approved by the Boston College Institutional Review Board (BC-IRB).

Participants were presented with the following scenario: “*You and a friend are standing in front of two buckets full of liquid*. *One bucket is full of very hot water*, *and one is full of a stranger’s urine*. *The urine is completely sterile*, *and the hot water is hot enough to be very painful but will not burn you*. *You each must dunk your hand in one bucket for 3 seconds*, *but you get to choose who gets which bucket*.”

Participants were randomly assigned to deliver judgments targeting one of four possible actions: (1) submerging one’s own hand in hot water, (2) submerging one’s own hand in sterile urine, (3) choosing for one’s friend to submerge his/her hand in hot water, or (4) choosing for one’s friend to submerge his/her hand in sterile urine. Each participant delivered one of three judgments for the target event: (1) how harmful, (2) how gross, or (3) how morally wrong is this, on a 7-point scale from “Not at All” to “Extremely”. Thus, each of the 526 participants delivered a single judgment for one of 12 different conditions (approximately 44 participants per condition).

### Results and discussion

As manipulation checks, we first conducted two separate 2 (target: self / other) x 2 (violation type: harm / purity) ANOVAs for judgments of harmfulness and grossness. We observed the predicted main effects of violation type (harmfulness: *F*(1,207) = 7.163, *p* = .008, partial η^2^ = .034; grossness: *F*(1,159) = 9.726, *p* = .002, partial η^2^ = .059): across targets, the harm violation (i.e., hand in hot water) was rated as more harmful (*M* = 4.41, *SD* = 1.85) than the purity violation (i.e., hand in urine; *M* = 3.66, *SD* = 2.12; *t*(206) = 2.72, *p* = 0.007), and the purity violation was rated as more gross (*M* = 4.62, *SD* = 2.08) than the harm violation (*M* = 3.52, *SD* = 2.29; *t*(158) = 3.17, *p* = 0.002). For judgments of grossness, we also found an unexpected main effect of target (*F*(1,159) = 8.723, *p* = .004, partial η^2^ = .053): violating another person (via harm or impurity) was rated as more gross than violating oneself (self: *M* = 3.59, *SD* = 2.24; other: *M* = 4.62, *SD* = 2.13; *t*(158) = 2.92, *p* = 0.004). Participants may have interpreted “gross” as morally abhorrent in addition to physically disgusting; in general, violating another person may be perceived as more morally abhorrent than violating the self [41]. There were no significant target x violation type interactions (*p*>0.40).

**Fig 1:**
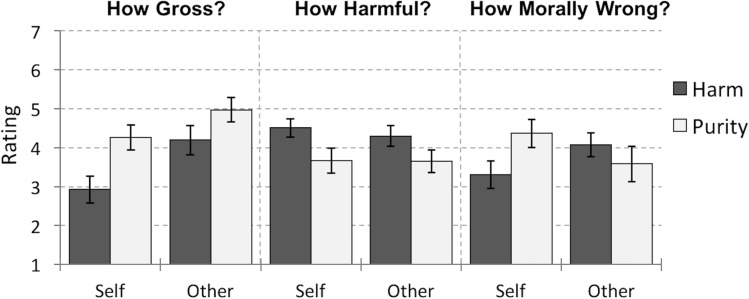
Three Judgments Made in Study 1. Ratings are broken down by violation type (harm versus purity) and target (violations targeting oneself versus another). Participants rated each question using a scale from 1 (Not at All) to 7 (Extremely). Error bars represent standard error.

For moral judgments, a 2 (target: self / other) x 2 (violation type: harm / purity) between-subjects ANOVA revealed no main effects of either target or violation type (*p*’s>0.40). Critically, however, we observed the key predicted interaction between target and violation type (*F*(1,157) = 4.352, *p* = .039, partial η^2^ = .027; Fig 1). For actions targeting oneself, the purity violation was rated as more morally wrong than the harm violation (harm: *M* = 3.31, *SD* = 2.28; purity: *M* = 4.37, *SD* = 2.30; *t*(81) = 2.10, *p* = 0.039); by contrast, for actions targeting another person, the opposite, though non-significant, pattern emerged: the harm violation was rated as more morally wrong than the purity violation (harm: *M* = 4.08, *SD* = 2.20; urine: *M* = 3.58, *SD* = 2.2; *t*(73) = 0.91, *p*>0.30).

These results provide partial support for our hypothesis. While, as expected, judgments of harmfulness and grossness tracked the content of a moral violation (harm versus purity), judgments of moral wrongness revealed an important interaction between content and target. However, this interaction was driven primarily by moral judgments of self-directed acts: participants rated defiling themselves (i.e., choosing the bucket with urine) as much worse than harming themselves (i.e., choosing the bucket with hot water), but rated defiling another person as similarly wrong compared to harming another person.

One potential concern with these results is that the scenario we used makes decisions for oneself and another person non-independent. In other words, defiling oneself by choosing the bucket with urine would necessarily mean harming another, because the friend would be left with the bucket of hot water. This forced-choice design leaves unknown whether participants deliver harsh judgments of defiling oneself because they are focused on choosing the impure act for themselves, or relegating the harmful act to their friend. To address this concern, Study 2 employed a design involving independent choices for all moral violations.

## Study 2: Testing a range of harm and purity violations

Study 2 expands on the results of Study 1 in several important ways. First, we decouple choices for oneself and another by presenting one group of participants with harm and purity violations targeting themselves, and a separate group of participants with harm and purity violations targeting another person. Additionally, we test a series of different harm and purity violations to ensure that our results generalize beyond the scenario used in Study 1. Finally, participants deliver three judgments of each violation, allowing us not only to validate that harm violations are seen as harmful and purity violations are seen as gross, as in Study 1, but also to test how ratings of harmfulness and grossness predict moral judgments across conditions.

### Method

Using MTurk, we collected data from an online sample of 150 American participants (65% male, *M*_age_ = 31.73, *SD*_age_ = 10.88). All participants gave written informed consent and were paid $0.76 for their time. All experimental procedures were approved by the BC-IRB. Twenty-two participants were excluded for failing to complete the survey.

Participants were presented with a list of twenty violations: ten harm violations (e.g., a pinprick on the hand, breaking a leg) and ten purity violations (e.g., cook and eat your pet dog, French-kissing a cousin). The items were designed to vary in severity, and the purity items in particular represented diverse purity concerns, including core disgust, sexual concerns, and interpersonal/moral contamination [7,31,42,43]. Participants were randomly assigned to one of two independent conditions in a between-subjects design: (1) rendering the *self* a victim of all violations, or (2) rendering *another person* (i.e., a friend) a victim of all violations (approximately 64 participants per condition).

Participants then delivered three judgments of all 20 violations: (1) how harmful, (2) how gross, and (3) how morally wrong it would be to choose each item for themselves or their friend. Each kind of judgment was presented in a separate block, with presentation order of the three blocks counterbalanced across participants. Participants used a 100-point sliding scale from “Not At All” to “Extremely” for all three sets of judgments.

### Results and discussion

As manipulation checks, we first conducted two separate 2 (target: self / other) x 2 (violation type: harm / purity) ANOVAs for judgments of harmfulness and grossness. We observed the predicted main effects of violation type (harmfulness: *F*(1,126) = 189.763, *p*<0.001, partial η^2^ = 0.601; grossness: *F*(1,126) = 284.289, *p*<0.001, partial η^2^ = 0.693): across targets, harm items were rated as more harmful (*M* = 54.42, *SD* = 17.84) than purity items (*M* = 27.75, *SD* = 20.93; *t*(127) = 13.813, *p*<0.001), and purity items were rated as more gross (*M* = 59.74, *SD* = 17.23) than harm items (*M* = 25.34, *SD* = 21.58; *t*(127) = 16.859, *p*<0.001). There were no significant effects of target or violation type x target interactions.

**Fig 2:**
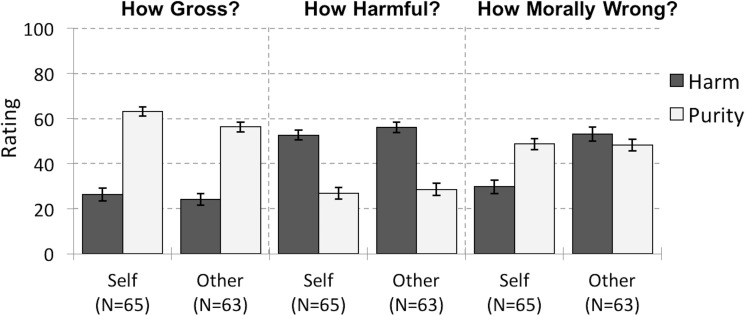
Three Judgments Made in Study 2. Ratings are broken down by violation type (harm / purity) and target (violations targeting oneself / another). Participants rated each question using a sliding scale from 1 (Not at All) to 100 (Extremely). Error bars represent standard error.

For moral judgments, a 2 (target: self / other) x 2 (violation type: harm / purity) ANOVA revealed a main effect of violation type (*F*(1,126) = 11.738, *p* = .001, partial η^2^ = .085). Participants rated purity violations as more morally wrong than harm violations (purity: *M* = 48.52, *SD* = 20.24; harm: *M* = 41.29, *SD* = 26.77). We also observed a main effect of target (*F*(1,126) = 11.584, *p* = .001, partial η^2^ = .084). Violations affecting others were rated as more morally wrong (*M* = 50.73, *SD* = 2.401) than violations affecting the self (*M* = 39.27, *SD* = 2.364). Most importantly, we again found the key predicted interaction between target and violation type (*F*(1,157) = 4.352, *p* = .039, partial η^2^ = .027; Fig 2). When rating violations affecting themselves, participants rated purity violations more morally wrong than harm violations (purity: *M* = 48.71, *SD* = 19.88; harm: *M* = 29.82, *SD* = 24.05; *t*(64) = 6.471, *p* < .001); by contrast, when violations targeted another person, the opposite though non-significant trend emerged: harm violations were judged more morally wrong than purity violations (harm: *M* = 53.13, *SD* = 24.29; purity: *M* = 48.33, *SD* = 20.77; *t*(62) = 1.658, *p* = .102). In addition to rating each violation, participants in this study rank-ordered all twenty violations from least morally wrong to most morally wrong. Analyses of these rankings replicated this key interaction between target and violation type and are presented in Supporting Information (S1 Text).

We also conducted a linear regression predicting judgments of moral wrongness from judgments of harmfulness, grossness, whether an action targeted oneself versus another, and their interactions (Table 1). Both judgments of harmfulness and grossness strongly predicted moral judgments: the more harmful and gross an action was, the more morally wrong participants rated the action. Actions targeting others were also rated as more morally wrong than actions targeting oneself. Most notably, judgments of harmfulness and grossness interacted with this factor (self versus other) differently: the more harmful an act was, the more morally wrong actions targeting others were rated, whereas the more gross an act was, the more morally wrong actions targeting oneself were rated.

**Table 1:** Results of linear regression analyses predicting moral judgment ratings (across both harm and purity violations) from ratings of harmfulness, grossness, whether an action targeted oneself versus another person (self = 1, other = 0), and interaction terms.

Predictors	Beta	*SE* (*B*)	*t*	*p*	Semi-partial correlation
How Harmful?	0.606	0.064	9.446	<0.001	0.417
How Gross?	0.298	0.062	4.814	<0.001	0.212
Target: (Self = 1; Other = 0)	-12.158	5.818	- 2.090	0.038	- 0.092
**How Harmful x Target**	**- 0.327**	**0.091**	**- 3.614**	**<0.001**	**- 0.159**
**How Gross x Target**	**0.312**	**0.083**	**3.771**	**<0.001**	**0.166**

These results replicate and extend our findings from Study 1. We again observed a strong effect whereby participants rated defiling themselves as more morally wrong than harming themselves. We observed the opposite trend for actions targeting others: participants rated harming others as more morally wrong than defiling others, though, as in Study 1, this difference was not significant. However, looking across harm and purity violations, ratings of harmfulness predicted harsher moral judgments of actions targeting others, whereas ratings of grossness predicted harsher moral judgments of actions targeting oneself. Together, these results provide further support for our hypothesis that purity norms govern the negative impact people have on themselves, whereas harm norms govern the negative impact people have on each other more broadly.

In both Studies 1 and 2, the participant was always the perpetrator of the moral violations. A consequence of this design choice is that victim status is confounded with whether the action was self-directed or dyadic: self-directed actions involved participants harming or defiling themselves, and dyadic actions involved participants harming or defiling other people. Study 3 systematically varies both the perpetrator and the victim of all violations, such that *self-directed* could also mean other people violating themselves and *dyadic* could also mean another person violating the participant. In addition to testing how our effects generalize across two additional relational contexts, this design allows us to test whether purity concerns emerge whenever the participant is the victim of another person’s impure action, or, alternatively, whenever an action is self-directed (regardless of whether or not the participant is involved).

## Study 3: Manipulating both the perpetrator and the victim

In Study 3, we manipulate both the perpetrator and the victim of violations to test 4 separate relational contexts: two self-directed (when either the participant or another person violates themselves) and two dyadic (when the participant violates another person, or another person violates the participant). We also include several additional measures. First, to address the possibility that the somewhat imprecise term “gross” was interpreted differently across participants (or as “morally abhorrent”, as suggested by the results of Study 1), we employ expanded descriptions, described below, in measuring judgments of harmfulness and impurity. Second, actions targeting others are presumably less consensual than actions targeting oneself, but it is unclear whether perceptions of consent differ across harm and purity violations. To this end, we measured perceptions of consent to ensure that they do not explain any observed differences between moral judgments of harm and purity violations. Finally, we again investigate how judgments of harm and impurity predict moral wrongness across relational contexts, as in Study 2, but include additional individual difference measures, including disgust sensitivity and scores on the Moral Foundations Questionnaire (MFQ) [7].

### Method

Using MTurk, we collected data from an online sample of 423 American participants (44% female, *M*_age_ = 32.7, *SD*_age_ = 11.5). All participants gave written informed consent and were paid $0.41 for their time. All experimental procedures were approved by the BC-IRB. Thirty participants were excluded for failing to recall the task they were asked to complete.

The survey depicted actions involving the participants themselves, using the second-person pronoun “you”, and other people, using the phrasing “someone else”, or “them”. The survey followed a 2 (violation type: harm / purity) x 2 (perpetrator: you / them) x 2 (target: you / them) between-subjects design. This design formed 8 conditions, whereby two different types of violations (harm / purity) occurred in 4 different relational contexts: 1) you violate yourself (“you-you”), 2) you violate another person (“you-them”), 3) someone else violates you (“them-you”), or 4) someone else violates themselves (“them-them”).

To further ensure that our results generalize across a range of violations considered to be harmful and impure, participants judged six examples of either harm or purity violations. Harm violations included physical harm (e.g., cutting someone on the arm), as well as emotional harm (e.g., calling someone fat and ugly) and property damage (e.g., destroying the money in someone’s wallet). Purity violations included pathogen threats (e.g., smearing cat poop on someone’s arm), sexually deviant acts (e.g., viewing a picture of bestiality), and sanctity offenses (e.g., selling someone’s soul to the devil). The order of violations was randomized across participants.

On one screen, participants delivered three judgments of each violation: 1) How morally wrong is this, 2) How harmful is this, and 3) How impure is this (order counterbalanced across participants). As mentioned above, given multiple interpretations of “harmful” and “impure”, we specified in each case that by harmful we meant how much pain and suffering the action causes, and by impure we meant how gross and unnatural was the action. On a subsequent screen, participants delivered a fourth judgment of how consensual they perceived each violation to be. All judgments were made on a 7-point Likert scale from “Not at All” to “Very”. Finally, participants answered demographic questions including political orientation (taken as the average of their political beliefs on social issues, economic issues, and overall; 7-point scales from “Very Liberal” to “Very Conservative”), a measure of trait disgust [5,44], and the MFQ [7].

### Results and discussion

For both harm and purity violations, we averaged ratings of the six items for each of the four judgments participants made (all α’s>0.82). As manipulation checks, we first conducted a series of 2 (violation type: harm / purity) x 2 (perpetrator: you / them) x 2 (target: you / them) ANOVAs to investigate how judgments of harm, impurity, and consent, differed across conditions. We again observed the predicted main effects of violation type for judgments of harmfulness (*F*(1,385) = 55.445, *p*<0.001, partial η^2^ = 0.126) and impurity (*F*(1,385) = 111.896, *p*<0.001, partial η^2^ = 0.225). Harm violations were rated as more harmful (*M* = 5.09, *SD* = 1.16) than purity violations (*M* = 4.14, *SD* = 1.60), though this difference was marginal in the “you-you” condition (*t*(99) = 1.898, *p* = .061; all other conditions *p*’s<0.001), consistent with previous research demonstrating that actions are perceived as less harmful in self-directed versus dyadic contexts [11]. Conversely, purity violations were rated as more impure (*M* = 5.70, *SD* = 1.27) than harm violations (*M* = 4.19, *SD* = 1.64), consistently across all four relational contexts (all *p*’s<0.005). Finally, for judgments of consent, dyadic acts were rated as less consensual (*M* = 1.52, *SD* = 1.01) than self-directed acts (*M* = 5.06, *SD* = 1.73; *t*(391) = 24.726, *p*<0.001). Most importantly, however, judgments of consent did not differ between harm violations (*M* = 3.35, *SD* = 2.29) and purity violations (*M* = 3.27, *SD* = 3.27) in any relational context (all *p*’s>0.24).

**Fig 3:**
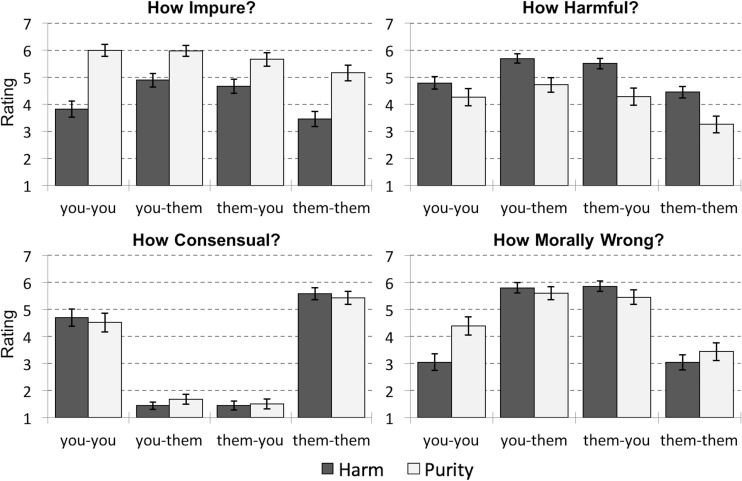
Four Judgments Made in Study 3. Ratings are broken down by violation type (harm / purity) and relational context (presented as: perpetrator-victim). Participants rated each question using a scale from 1 (Not at All) to 7 (Very). Error bars represent standard error.

We next performed the same 2 (violation type: harm / purity) x 2 (perpetrator: you / them) x 2 (target: you / them) ANOVA for judgments of moral wrongness. No main effects reached significance (all *p*’s>0.05). A significant perpetrator x target interaction indicated that violations in dyadic contexts (you-them and them-you) were judged as more morally wrong (*M* = 5.68, *SD* = 1.19) than self-directed violations (you-you and them-them; *M* = 3.48, *SD* = 1.80; *F*(1,385) = 215.612, *p*<0.001, partial η^2^ = 0.359). Furthermore, we observed a significant three-way interaction between violation type, perpetrator, and target (*F*(1,385) = 15.362, *p*<0.001, partial η^2^ = 0.038). Unpacking this interaction, in dyadic contexts, harm violations were rated as marginally more morally wrong (*M* = 5.83, *SD* = 0.99) than purity violations (*M* = 5.53, *SD* = 1.34; *t*(192) = 1.769, *p* = 0.079); however, in self-directed contexts, purity violations were rated as more morally wrong (*M* = 3.93, *SD* = 1.82) than harm violations (*M* = 3.05, *SD* = 1.68; *t*(197) = 3.573, *p*<0.001; Fig 3). This effect was driven by the context in which participants imagined acting on themselves (you-you; purity: *M* = 4.39, *SD* = 1.75; harm: *M* = 3.06, *SD* = 1.87; *t*(99) = 3.708, *p*<0.001).

As in Study 2, we also investigated how judgments of harm and impurity, among other factors, predicted moral judgments differently across each relational context. We first conducted four separate linear regressions predicting moral wrongness in each relational context from judgments of harmfulness, impurity, and consent, as well as trait disgust, political orientation, and scores on the harm and purity subscales of the MFQ. Judgments of consent and measures of trait disgust, political orientation, and MFQ_Harm scores did not predict judgments of moral wrongness in any relational context. Judgments of harmfulness and impurity again strongly predicted judgments of moral wrongness: in all four relational contexts, the more harmful and impure an action was, the more morally wrong participants rated it (Table 2). Interestingly, how harmful an action was predicted moral wrongness better than how impure an action was for both dyadic contexts (you-them and them-you), but the opposite pattern emerged for self-directed acts: that is, in both self-directed contexts (you-you and them-them), how impure an action was predicted moral wrongness better than how harmful. MFQ_Purity scores also predicted moral wrongness for self-directed acts (marginally in the you-you context), consistent with a larger role for purity in moral judgments of self-directed acts [5,11].

**Table 2:** Results of linear regression analyses predicting moral judgment in each relational context. Judgments of consent and measures of trait disgust, political orientation, and MFQ_Harm scores did not predict moral judgments in any relational context (all *p*’s>0.10).

Relational Context	Predictors	Beta	*SE* (*B*)	*t*	*p*	Semi-partial correlation
You—You	How Impure?	0.553	0.081	6.788	<0.001	0.417
	How Harmful?	0.471	0.092	5.137	<0.001	0.316
	MFQ_Purity	0.234	0.124	1.885	0.063	0.116
Them—Them	How Impure?	0.457	0.064	7.097	<0.001	0.432
	How Harmful?	0.423	0.080	5.259	<0.001	0.320
	MFQ_Purity	0.256	0.097	2.653	0.009	0.161
You—Them	How Harmful?	0.513	0.068	7.595	<0.001	0.519
	How Impure?	0.273	0.066	4.134	<0.001	0.282
Them—You	How Harmful?	0.496	0.062	8.043	<0.001	0.520
	How Impure?	0.274	0.064	4.280	<0.001	0.277

To further explore the impact harmfulness and impurity have on judgments of moral wrongness across self-directed versus dyadic contexts, we once again regressed judgments of moral wrongness onto judgments of harmfulness and impurity; however, we included whether the action was self-directed (versus dyadic) in the regression and probed interaction effects between the factors. Judgments of both harm and impurity, as well as whether or not an action was self-directed strongly predicted judgments of moral wrongness (Table 3). We found no interaction between judgments of harmfulness and whether an act was self-directed versus dyadic. In other words, judgments of how harmful an act was predicted moral judgment to a similar extent for both self-directed and dyadic contexts, consistent with recent work suggesting that harm plays a primary role in moral judgments across domains [41,45]. Critically, however, we did observe a significant interaction between judgments of impurity and whether or not an act was self-directed. How impure an act was predicted harsher moral judgments to a greater extent for self-directed acts relative to dyadic acts.

**Table 3:** Results of a linear regression analysis predicting moral judgment from judgments of harm, impurity, the action’s relational context (self-directed = 1, dyadic = 0), and interaction effects.

Predictors	Beta	*SE* (*B*)	*t*	*p*	Semi-partial correlation
How Harmful?	0.510	0.052	9.855	<0.001	0.252
How Impure?	0.278	0.050	5.596	<0.001	0.143
Relational Context (Self-directed = 1, Dyadic = 0)	-1.754	0.122	-14.405	<0.001	-0.368
How Harmful x Relational Context	0.007	0.071	0.100	0.920	0.003
**How Impure x Relational Context**	**0.306**	**0.064**	**4.793**	**<0.001**	**0.123**

These patterns of moral judgment clarify the results of Studies 1 and 2 and lend further support for a key aspect of our hypothesis. Specifically, purity violations were again rated as more morally wrong than harm violations when perpetrators violate themselves, and ratings of how impure an action was predicted moral judgments more in these self-directed contexts than in dyadic contexts. By contrast, harm violations were rated as only marginally more wrong than purity violations when a perpetrator violates another person, and judgments of how harmful an action was predicted moral judgments similarly across self-directed and dyadic contexts. Interestingly, these results suggest that purity concerns do not emerge whenever a person is the victim of an impure action (as in the dyadic “them-you” condition), but rather whenever people defile themselves (both self-directed contexts “you-you” and “them-them”). Together, these results illuminate how the relational context of an action interacts with its content to determine moral judgment.

## General discussion

Recent taxonomies of moral psychology focus primarily on either the specific content of moral actions or their relational context. Moral Foundations Theory suggests that moral psychology may be divided into at least five domains: harm, fairness, loyalty, respect, and purity [7,46]. These domains are largely defined by their descriptive content; for example, assaulting a person belongs to the harm domain, while taking unfair advantage of a person belongs to the fairness domain. An alternative account emphasizes the influence of the relational context of an action, i.e. not paying for a meal is considered stealing at a restaurant, while paying for a meal is considered rude at your grandmother’s house [9,10,47]. On this context-driven account, how we perceive an act and its moral status depends primarily on the identities of the parties involved and, importantly, their relationship. The current results suggest a compromise: moral judgments are best characterized by considering both the content (e.g., harm versus purity) and the relational context (e.g., self-directed versus dyadic) of moral actions.

In three studies, we demonstrated that moral judgments reflect an interaction between an action’s content and its relational context. Across a wide range of violations, participants judged defiling oneself as more morally wrong than harming oneself, but judged defiling others equally as wrong as harming others. Moreover, perceptions of impurity predicted moral judgments more when someone violated themselves than when someone violated another person, whereas perceptions of harm predicted moral judgments to a similar extent across all relational contexts. Together, these results support one key aspect of our hypothesis: that purity norms track the negative impact people have on themselves.

This finding may provide insight into the adaptive function of purity norms. Dominant theories in evolutionary and moral psychology suggest purity norms stem from disgust reactions to substances or behaviors that signal disease and contamination [6,27,34]. However, if purity norms are in place simply to protect people from harmful pathogens, it is unclear why in the current data perceptions of impurity predicted moral judgments more for self-directed, actions (whether the actor was oneself or another) than dyadic actions; notably, including when the participant was the victim of another person’s impure action. In other words, perceptions of impurity did not predict moral judgments simply when participants themselves were the victims of disgusting actions, but rather when people violated themselves. Thus, while pathogen concerns likely elicit emotional reactions of disgust, they do not seem to be the primary motivator of moral judgments of purity violations.

We suggest an alternative account: purity norms evolved to help establish group boundaries by identifying people that behave similarly and conform to group norms (e.g., ingroup members). Purity violators are seen as abnormal [20] and tagging deviant behavior as offensive may help define group norms and establish cultural boundaries [48,49] by increasing behavioral homogeneity [12]. While certain behaviors may cause group members to gain or lose status within their social group, behaviors that conform to or violate purity norms may serve as a coarser signal of whether someone belongs to a group in the first place. Consistent with this account is other recent work showing that purity norms predict how close people are in a social network better than other moral convictions [50]. Even non-pathogen based concerns, such as flag-burning and global warming, are associated with people’s purity values in the context of taking sides in America’s political “culture war” [51]. Relating this work to our present findings, how people treat themselves–what they eat, how they treat their bodies, or the people they associate with–may be more indicative of a person’s social identity than how they treat others.

One potential concern with this account is its blurring of the distinction between moral norms and social or conventional norms. While our participants consistently rated the moral wrongness of diverse purity violations above floor, purity norms may nevertheless be closer to social conventions in important respects than harm norms. As the current evidence suggests, harm norms apply to prototypical moral situations involving a violator and a victim [41], whereas purity norms apply primarily to situations involving individuals acting on themselves. Groups may have more flexibility in establishing norms for how ingroup members conduct themselves compared to how people treat each other. Consistent with this, culturally-specific conceptions of the self lead to robust variation in the moral status of purity norms compared to relatively stable endorsement of harm norms [52,53]. Even groups within the United States (i.e., liberals and conservatives) differ in their endorsement of purity but not harm norms [7,44]. Future work can characterize how social, emotional, and cultural factors interact to determine when and how purity violations are moralized.

The present account of purity norms also aligns well with existing evidence for distinct cognitive processing of harm and purity violations. First, information about a violator’s intentions influence moral judgments of harm violations more than purity violations [11,12,18]. In the current framework, intentions may exert relatively little influence on judgments of purity violations since actions alone are often sufficient to signal group membership (e.g., Does the violator follow the ingroup’s customs or not?) [12,18]. In contrast, when people evaluate a violator’s harmful actions toward a victim, they rely on information about the violator’s intent to determine whether the violator is likely to cause harm again (e.g., Is the violator likely to be friend or foe?), as intentions afford reliable predictions of future behavior [12,54]. Second, purity violations, more so than harm violations, are attributed to dispositional versus situational factors [20] and strongly affect perceptions of the violator’s moral character [55,56]. While prior work has focused on moral character as a global evaluation of a person’s moral standing [57,58], purity violations may reveal specific information about an individual’s group status. In other words, we suggest that, while a harm violator may be seen as a worse *person*, a purity violator may be seen as a worse *group member*. Future work should further investigate whether these two aspects of character dissociate for moral evaluations of blame and punishment, as well as other social evaluations such as choosing partners or teammates. Indeed, the qualities that make a group member valuable are often distinct from moral qualities in the abstract (e.g., whistleblowers, who may be seen as upstanding moral individuals, are often derided and ostracized by loyal group members) [59].

We found limited evidence of a specific link between moral judgments of harm violations and the negative impact actions have on others. Across three studies, people rated harm and purity violations as equally morally wrong when targeting others. Despite our manipulation checks, this may be partly because both harm and purity violations are perceived as relatively more harmful when directed at others [11]. Study 2 did find that ratings of harmfulness predicted moral judgments more for other-directed versus self-directed acts; however, this effect did not replicate in Study 3, where perceptions of harm predicted judgments of both dyadic and self-directed actions to a similar extent. Though inconsistent with our initial hypothesis, these findings are consistent with work showing that concerns about harm play a predominant role in most Americans’ moral psychology [41,60]. Harm norms are more universally held than purity norms [39,53], and people may be prone to seeing any negative dyadic interaction through the lens of a perpetrator harming a victim [45]. Future studies should investigate whether harm does indeed have a more specific link with the impact an action has on others (versus oneself) in populations that are less harm-focused, such as non-Western societies [52,53,61].

Recent work has argued that purity violations actually boil down to harm violations [45,60]. Given that purity violations may impact perceptions of violators’ character and group status, subsequent judgments may in the abstract reflect consideration of a particular kind of harm: social harm. However, we note that, at least in the present data, judgments of harmfulness dissociated from judgments of moral wrongness. Moreover, if harm considerations in general motivated moral judgments, the immediate damage caused by harm violations might represent greater threat than potential harm caused by purity violations. Instead, we consistently observed harsher moral judgments of self-directed purity violations compared to self-directed harm violations. Thus, it does not seem to be the case that participants are simply basing their moral judgments (of all kinds of violations) on perceptions of harm.

While we have focused here on the distinction between harm and purity norms, a primary question for future research is whether the differences observed across self-directed and dyadic contexts applies to other moral norms described by theories such as Moral Foundations Theory, including loyalty, hierarchy, and fairness [26]. Concerns about purity, loyalty, and hierarchy are said to reflect “binding norms”, in that they bind people into cooperative and cohesive groups, whereas concerns about harm and fairness reflect “individualizing norms”, in that they govern how people interact as individuals, within and across group boundaries [6,7]. As such, binding norms may protect the self, insofar as the group provides people with protective benefits (including in terms of social reputation), whereas individual norms may apply more broadly to people’s interactions with others. Intriguingly, while there is little empirical work on the cognitive inputs to judgments of loyalty, hierarchy, and fairness violations, judgments of binding versus individualizing norms may reflect the same cognitive differences observed between purity and harm violations; in particular, the greater role of outcomes relative to other contextual features [62–64]. Future research should test whether judgments of other moral domains also track with concerns for different relational contexts, as outlined here [65]. Doing so will provide a more detailed characterization of moral judgments and may inform current theories on the adaptive functions of distinct moral norms.

## Supporting information

S1 Appendix Stimuli and Measures. Stimuli and dependent measures for all studies are reported in full.
(DOCX)

S1 Text Analyses of Ranking Data. In the main text, we report an interaction between target (self / other) and violation type (harm / purity) for judgments of moral wrongness. Here, we report results replicating this interaction when participants rank-ordered all violations from least morally wrong to most morally wrong.
(DOCX)
