# Supplementary material for: The relevance of moral norms in distinct relational contexts: Purity versus harm norms regulate self-directed actions
Source: PLoS One. 2017 Mar 9;12(3):e0173405. doi: 10.1371/journal.pone.0173405 (PMC5344389; doi:10.1371/journal.pone.0173405)
Supplement: S1 Appendix — Stimuli and dependent measures for all studies are reported in full. (DOCX) [file pone.0173405.s001.docx]

**S1 Appendix: Stimuli and Measures**

**Study 1**

All participants saw the following vignette:

“*You and a friend are standing in front of two buckets full of liquid. One bucket is full of very hot water, and one is full of a stranger’s urine. The urine is completely sterile, and the hot water is hot enough to be very painful but will not burn you. You each must dunk your hand in one bucket for 3 seconds, but you get to choose who gets which bucket.*”

Participants delivered judgments about one of four possible actions:

“Imagine that you choose to submerge your…”

1. …own hand in the urine.
2. …friend’s hand in the urine.
3. …hand in the water.
4. …friend’s hand in the water.

Each participant delivered one of three judgments of the action they saw using a 7-point scale from “Not at All” to “Extremely”:

1. How harmful is this?
2. How gross is this?
3. How morally wrong is this?

**Study 2**

Participants were randomly assigned to one of two independent conditions: 1) rendering themselves a victim of all violations, or 2) rendering another person a victim of all violations. The instructions were as follows:

For the following list of things, imagine each of them happening to **(you / your friend)**. Imagine that you must choose what will happen to **(you / them)** among these items.

Ranking Task:

All participants were first instructed to rank each item (1-20) from:

1) The LEAST morally wrong thing to do to **(yourself / your friend)**, to

20) The MOST morally wrong thing to do to **(yourself / your friend)**.

Rating Task:

All participants then delivered three judgments of all 20 violations: (1) how harmful, (2) how gross, and (3) how morally wrong it would be to choose each item for themselves or their friend. Each kind of judgment was presented in a separate block, with presentation order of the three blocks counterbalanced across participants. Participants used a 100-point sliding scale from “Not At All” to “Extremely” for all three sets of judgments. Instructions were as follows:

Again, imagine that you must choose what will happen to (you/your friend) among these items.

Rate how morally wrong it would be for you to choose each item for (yourself / your friend) from 0 (Not at All Wrong), to 100 (Extremely Wrong).

Rate how harmful it would be for you to choose each item for (yourself / your friend) from 0 (Not at All Harmful), to 100 (Extremely Harmful).

Rate how gross it would be for you to choose each item for (yourself / your friend) from 0 (Not at All Gross), to 100 (Extremely Gross).

Harm Items:

(Your / Their) cheek is pinched.

(You / They) get knocked down on concrete.

(Your / Their) hand is pricked with a needle.

(You / They) receive a painful electric shock.

(Your / Their) arm is cut with a small knife.

(Your / Their) finger gets smashed with a hammer.

(You / They) get kicked in the head, hard.

(You / They) get shot with a pellet gun.

(You / They) get sprayed in the eyes with mace.

(You / They) get (your / their) leg broken.

Purity Items:

(You / They) must lick between (your / their) toes.

(You / They) must pick up dog poop barehanded.

(You / They) must step in vomit barefoot.

(You / They) must French-kiss (your / their) first-cousin.

(You / They) must touch the genitals of a human corpse.

(You / They) must cook and eat (your / their) pet dog after it dies of natural causes.

(You / They) must get a blood transfusion of disease-free blood from a convicted child

molester.

(You / They) must get saline injections to temporarily give (your / their) forehead horns.

(You / They) must sign a piece of paper selling (your / their) soul to the devil.

(You / They) must spend a day wearing a sweater owned by Adolf Hitler.

Attention Check, asked at the end of the study:

Please describe the situation you were first told to imagine when ranking things.

**Study 3**

Participants were assigned to one of eight possible conditions in a 2 (violation type: harm / purity) x 2 (perpetrator: you / them) x 2 (target: you / them) between-subjects design. The survey depicted actions involving the participants themselves, using the second-person pronoun “you”, and other people, using the phrasing “someone else”, or “them”. Thus, harm and purity violations occurred in four possible relational contexts: 1) you violate yourself (“you-you”), 2) you violate another person (“you-them”), 3) someone else violates you (“them-you”), or 4) someone else violates themselves (“them-them”).

Harm Violations:

1. Imagine that (you / someone) punches (you / them) in the ribs.
2. Imagine that (you / someone) cuts (you / them) on the arm.
3. Imagine that (you / someone) pours scalding hot water on (your / their) lap.
4. Imagine that (you / someone) deprives (you / them) of food for 2 days.
5. Imagine that (you / someone) calls (you / them) fat and ugly when (you / they) are trying on new jeans.
6. Imagine that (you / someone) destroys all the money in (your / their) wallet.

Purity Violations:

1. Imagine that (you / someone) buys dog meat and serves it to (you / them) for lunch.
2. Imagine that (you / someone) smears cat poop on (your / their) arm.
3. Imagine that (you / someone) pours urine on (your / their) lap.
4. Imagine that (you / someone) touches a used condom to (your / their) face.
5. Imagine that (you / someone) changes (your / their) phone background to a picture of a man having sex with a horse.
6. Imagine that (you / someone) offers (your / their) soul to the devil as part of a ritual sacrifice.

All participants delivered 4 judgments of each scenario using 7-point scales from “Not at All” to “Very”. The order of the first three judgments was counterbalanced across participants. Judgments of consent were presented last on a different screen.

1. How morally wrong is this?
2. How harmful is this (by harmful we mean how much pain and suffering does it cause)?
3. How impure is this (by impure we mean how gross and unnatural is it)?
4. How consensual do you think this was?

Attention Check:

Please describe one of the scenarios you were told to imagine.

Participants indicated their political orientation by answering the following 3 questions using a 7-point scale from 1 (Liberal) to 7 (Conservative):

Using the scale below, please describe your political beliefs:

1. On social issues:
2. On fiscal issues:
3. Overall:

As a measure of trait disgust, participants completed part of the Disgust Sensitivity Scale, Version 2 (Haidt, McCauley, & Rozin, 1994). Participants only responded to the eight items measuring core disgust (as in: Inbar, Pizarro, & Bloom, 2009; Rottman, Kelemen, & Young, 2014):

Please indicate how much you agree with each of the following statements, or how true it is about you.

1. I might be willing to try eating monkey meat, under some circumstances.

2. If I see someone vomit, it makes me sick to my stomach.

3. Seeing a cockroach in someone else’s house does not bother me. (Reverse coded)

4. Even if I was hungry, I would not drink a bowl of my favorite soup if it had been stirred by a used but thoroughly washed fly swatter.

How disgusting would you find each of the following experiences?

5. You see maggots on a piece of meat in an outdoor garbage pail.

6. While you are walking through a tunnel under a railroad track, you smell urine.

7. A friend offers you a piece of chocolate shaped like dog-doo.

8. You see a bowel movement left unflushed in a public toilet.

Citation: Haidt J, McCauley C, Rozin P. Individual differences in sensitivity to disgust: A scale sampling seven domains of disgust elicitors. Pers Individ Dif. 1994;16(5):701-713.

Participants also completed The Moral Foundations Questionnaire (full version, July 2008) by Jesse Graham, Jonathan Haidt, and Brian Nosek, downloaded from [www.MoralFoundations.org](http://www.MoralFoundations.org)

When you decide whether something is right or wrong, to what extent are the following considerations relevant to your thinking? Please rate each statement using this scale:

[0] = not at all relevant (This consideration has nothing to do with my judgments of right and wrong)

[1] = not very relevant

[2] = slightly relevant

[3] = somewhat relevant

[4] = very relevant

[5] = extremely relevant (This is one of the most important factors when I judge right and wrong)

______Whether or not someone suffered emotionally

______Whether or not some people were treated differently than others

______Whether or not someone’s action showed love for his or her country

______Whether or not someone showed a lack of respect for authority

______Whether or not someone violated standards of purity and decency

______Whether or not someone was good at math

______Whether or not someone cared for someone weak or vulnerable

______Whether or not someone acted unfairly

______Whether or not someone did something to betray his or her group

______Whether or not someone conformed to the traditions of society

______Whether or not someone did something disgusting

______Whether or not someone was cruel

______Whether or not someone was denied his or her rights

______Whether or not someone showed a lack of loyalty

______Whether or not an action caused chaos or disorder

______Whether or not someone acted in a way that God would approve of

Part 2. Please read the following sentences and indicate your agreement or disagreement:

[0] [1] [2] [3] [4] [5]

Strongly Moderately Slightly Slightly Moderately Strongly

disagree disagree disagree agree agree agree

______Compassion for those who are suffering is the most crucial virtue.

______When the government makes laws, the number one principle should be ensuring that everyone is treated fairly.

______I am proud of my country’s history.

______Respect for authority is something all children need to learn.

______People should not do things that are disgusting, even if no one is harmed.

______It is better to do good than to do bad.

______One of the worst things a person could do is hurt a defenseless animal.

______Justice is the most important requirement for a society.

______People should be loyal to their family members, even when they have done something wrong.

______Men and women each have different roles to play in society.

______I would call some acts wrong on the grounds that they are unnatural.

______It can never be right to kill a human being.

______I think it’s morally wrong that rich children inherit a lot of money while poor children inherit nothing.

______It is more important to be a team player than to express oneself.

______If I were a soldier and disagreed with my commanding officer’s orders, I would obey anyway because that is my duty.

______Chastity is an important and valuable virtue.
