# Supplementary material for: The relevance of moral norms in distinct relational contexts: Purity versus harm norms regulate self-directed actions
Source: PLoS One. 2017 Mar 9;12(3):e0173405. doi: 10.1371/journal.pone.0173405 (PMC5344389; doi:10.1371/journal.pone.0173405)
Supplement: S1 Text — In the main text, we report an interaction between target (self / other) and violation type (harm / purity) for judgments of moral wrongness. Here, we report results replicating this interaction when participants rank-ordered all violations from least morally wrong to most morally wrong. (DOCX) [file pone.0173405.s002.docx]

**S1 Text: Analyses of Ranking Data**

In addition to rating each violation, participants from Study 2 in both target-conditions (self and other) also rank-ordered all twenty violations from least morally wrong (1) to most morally wrong (20). We present a replication of the key target x violation type interaction in Study 2 using this additional ranking data from the same participants.

Using standard ANOVAs to test for an interaction is inappropriate given the non-independence of the rank data; however, for ease of interpretation, we first present a 2 (target: self versus other) x 2 (violation type: harm versus purity) mixed-effects ANOVA of participants’ average ranking of all 10 harm violations compared to their average ranking of all 10 purity violations. This revealed the predicted interaction between target and violation type (F(1,126)=5.031, p=.027, partial η^2^ =.038) in the absence of any main effects (*p*’s>.05).

Although Freedman's test could be used to compare the ranks of two types of items from multiple individuals, it cannot test for the interaction between violation type and target; therefore, we used a novel rank-based nonparametric method (Wu, in prep). This method estimates the probability that a randomly chosen participant from each target-condition (self versus other) rates a randomly chosen harm violation as less preferable than a randomly chosen purity violation. A probability greater than 0.5 indicates that, on average, participants in this target-condition rate a given harm violation as preferable to a purity violation. A difference in this probability across the two targets indicates an interaction between target and violation type.

In line with our prediction and consistent with the prior analyses, the probabilities across the two targets (P_1_ = ranking for others, P_2_ = ranking for self) are significantly different (P_1_=0.589; P_2_=0.640; z=2.25, two-sided *p*=0.025, odds ratio =1.24, 95% CI (1.03, 1.50)). Participants ranking for themselves are significantly more likely to prefer harm versus purity violations (z=2.608, *p*=.009), compared to participants ranking for others (z=1.255, *p*=.209). Thus, ranking data replicates the target x violation type interaction for participant’s ratings reported in Study 2. Impure actions are ranked as more morally wrong than harmful actions when the victim is oneself. When the victim is another person, this ranking changes significantly: harmful actions are ranked as more morally wrong than impure actions, though as in Studies 1 and 2 in the main text, this difference was not significant.
